# Interpreting the pathogenicity of Joubert syndrome missense variants in *Caenorhabditis elegans*

**DOI:** 10.1242/dmm.046631

**Published:** 2021-01-26

**Authors:** Karen I. Lange, Sofia Tsiropoulou, Katarzyna Kucharska, Oliver E. Blacque

**Affiliations:** School of Biomolecular and Biomedical Science, Conway Institute, University College Dublin, Dublin 4, Ireland

**Keywords:** B9D2, *C. elegans*, Joubert syndrome, Cilia, MKSR-2, Transition zone

## Abstract

Ciliopathies are inherited disorders caused by defects in motile and non-motile (primary) cilia. Ciliopathy syndromes and associated gene variants are often highly pleiotropic and represent exemplars for interrogating genotype-phenotype correlations. Towards understanding disease mechanisms in the context of ciliopathy mutations, we have used a leading model organism for cilia and ciliopathy research, *Caenorhabditis elegans*, together with gene editing, to characterise two missense variants (P74S and G155S) in *mksr-2/B9D2* associated with Joubert syndrome (JBTS). B9D2 functions within the Meckel syndrome (MKS) module at the ciliary base transition zone (TZ) compartment and regulates the molecular composition and sensory/signalling functions of the cilium. Quantitative assays of cilium/TZ structure and function, together with knock-in reporters, confirm that both variant alleles are pathogenic in worms. G155S causes a more severe overall phenotype and disrupts endogenous MKSR-2 organisation at the TZ. Recapitulation of the patient biallelic genotype shows that compound heterozygous worms phenocopy worms homozygous for P74S. The P74S and G155S alleles also reveal evidence of a very close functional association between the B9D2-associated B9 complex and MKS-2/TMEM216. Together, these data establish *C. elegans* as a model for interpreting JBTS mutations and provide further insight into MKS module organisation.

This article has an associated First Person interview with the first author of the paper.

## Abstract (precis)

**Editor’s choice:** Genome editing was carried out in *C. elegans* to model and characterise two pathogenic missense variants of *mksr-2/B9D2*, P74S and G155S, from a compound heterozygous patient with Joubert syndrome.

## INTRODUCTION

Ciliopathy disorders affect the development and/or homeostasis of many body tissues and organs and consist of at least 35 clinically distinct genetically inherited diseases. Examples include Joubert syndrome (JBTS), Meckel syndrome (MKS) and nephronophthisis (NPHP), which present with overlapping clinical presentations including brain malformations, bone abnormalities, sensory defects, left-right asymmetries, cystic kidney disease, retinal degeneration and cognitive impairment (Wheway et al., 2019). Ciliopathies result from defects in motile or primary cilia that extend from the surfaces of most eukaryotic cell types. Non-motile primary cilia function as antenna-like organelles, responding to many extracellular sensory cues, such as light, odorants and osmotic strength, in addition to cell-cell signalling molecules (e.g. sonic hedgehog) that regulate cell behaviour, tissue formation and homeostasis (Anvarian et al., 2019). The canonical primary cilium consists of a cylinder of nine doublet microtubules extending from a plasma membrane-anchored basal body (Satir et al., 2010).

Despite the cytosol and membrane of the cilium being contiguous with those of the cell, the organelle is highly compartmentalised, with a unique molecular composition and signalling environment. Compartmentalisation is achieved by active transport pathways and gating mechanisms at the ciliary base (Jensen and Leroux, 2017; Nachury and Mick, 2019). A major component of the ciliary gate is the proximal-most 0.2-1.0 µm of the axoneme, termed the transition zone (TZ). Characterised by Y-links that connect the ciliary membrane and axonemal microtubules, the TZ is thought to serve as a membrane- and size-dependent cytosolic permeability or diffusion barrier (Garcia-Gonzalo and Reiter, 2017; Reiter et al., 2012). At least 28 ciliopathy proteins localise at the TZ to regulate ciliary gating (Garcia-Gonzalo and Reiter, 2017; Reiter and Leroux, 2017). Mutations in TZ proteins disrupt the ciliary localisation of important signalling molecules, such as the Shh pathway regulator smoothened (Garcia-Gonzalo et al., 2011; Okazaki et al., 2020; Shi et al., 2017; Shimada et al., 2017).

TZ structure, function and molecular organisation are relatively well understood in *Caenorhabditis elegans*, in which cilia extend from the ends of sensory neuron dendrites (Inglis et al., 2007). Many ciliopathy proteins and associated pathways are conserved in the worm (Kim et al., 2018; van Dam et al., 2019), and studies of *C. elegans* have led to seminal discoveries that have greatly improved our understanding of conserved cilia biology. Genetic studies in *C. elegans* have revealed that TZ proteins function within two distinct entities, termed the ‘NPHP’ and ‘MKS’ modules (Williams et al., 2008, 2011). The *C. elegans* NPHP module comprises two proteins (NPHP-1/4), whereas the MKS module is larger and is subdivided into core (MKS-1/2, MKSR-1/2 and TMEM-231), intermediate (TMEM-107) and peripheral (TMEM-17/218, MKS-3/6 and JBTS-14) submodules that assemble at the TZ in a hierarchical fashion (Li et al., 2016; Williams et al., 2011). TZ recruitment of the entire MKS module depends on CEP-290 (Li et al., 2016; Schouteden et al., 2015). Additionally, MKS-5 (RPGRIP1L) recruits the NPHP and MKS modules, in addition to CEP-290, to the TZ and is therefore the master regulator of TZ assembly in *C. elegans* (Jensen et al., 2015). Consistent with a role in ciliary membrane gating, loss of individual MKS/NPHP module genes causes abnormal ‘leakage’ of membrane proteins into and out of cilia (Cevik et al., 2013; Williams et al., 2011). With regard to ciliogenesis, however, the NPHP and MKS modules function redundantly, with defects being observed only when a gene from both modules is disrupted (Williams et al., 2008, 2011). Although many aspects of NPHP/MKS modular organisation and assembly are conserved between worms and mammals (reviewed by Garcia-Gonzalo and Reiter, 2017; Reiter et al., 2012), including genetic interactions between the MKS and NPHP modules (Yee et al., 2015), there are some important differences. Notably, in mice, CEP290 regulates recruitment of the NPHP module, and RPGRIP1L interacts differentially with a paralogue (RPGRIP1) not present in worms to enable cell-specific mechanisms of TZ assembly (Wiegering et al., 2018).

Ciliopathy disorders are exemplars for interrogating human genotype-phenotype correlation, because the associated genes are frequently pleiotropic, with high levels of variability in their phenotypic expressivity. Despite this, most studies examine ciliopathy mechanisms using null allele, gene depletion or complementation-based approaches. Although important for revealing disease aetiology, these approaches do not address disease mechanisms directly in the context of specific genetic variants as they occur in patients. To address this issue, we have explored the use of *C. elegans* to model and interpret missense mutations associated with a TZ-associated ciliopathy. Specifically, we have focused on the TZ gene *B9D2*, linked to both Joubert and Meckel syndromes (JBTS34 and MKS10; MIM#614175) (Bachmann-Gagescu et al., 2015; Bader et al., 2016; Cui et al., 2011; Dowdle et al., 2011; Romani et al., 2014; Slaats et al., 2016). B9D2 is one of three MKS module proteins with a B9 domain, which is related to the C2 phospholipid binding domain and found only in ciliated eukaryotes (Zhang and Aravind, 2010). These three proteins (MKS1, B9D1 and B9D2) form the so-called B9 complex and localise to the TZ in an interdependent manner (Okazaki et al., 2020; Williams et al., 2008, 2011). As in *C. elegans*, B9D2 regulates ciliary gating in mouse, *Drosophila* and human cells (Basiri et al., 2014; Chih et al., 2012; Okazaki et al., 2020; Williams et al., 2011). B9D2 is also required for ciliogenesis in multiple organisms, including the mouse (Town et al., 2008) and *Paramecium* (Ponsard et al., 2007).

In this study, we have modelled two missense alleles (P74S and G155S) in B9D2, previously reported in a patient with JBTS (Bachmann-Gagescu et al., 2015). Using CRISPR-Cas9 genome editing, *C. elegans* alleles carrying the P74S and G155S mutations in the homologous positions of *C. elegans* *B9D2* (*mksr-2*) were generated. We found that endogenously expressed reporters for both variants localised to the TZ with reduced efficiency, with the G155S variant exhibiting a more severe reduction and a strikingly altered TZ distribution when visualised using super-resolution microscopy. Quantitative assays of cilium structure, sensory function and ciliary gating identified that the G155S variant exhibited more severe phenotypes than P74S. Both variants also somewhat disrupt the TZ recruitment of MKS module proteins, with the effect on MKS-2 being especially prominent. Lastly, compound heterozygous worms (P74S/G155S), which reflect the biallelic genotype of the JBTS patient, display a phenotype approaching that of worms recessive for the P74S mutation. Taken together, these data demonstrate the utility of *C. elegans* for interpreting the pathogenicity of missense alleles on B9D2 function. Our work also uncovers new insight into MKS module organisation at the TZ, including a close functional association between the B9 complex and MKS-2/TMEM216.

## RESULTS

### Modelling pathogenic missense B9D2 variants associated with Joubert syndrome

**Fig. 1.**
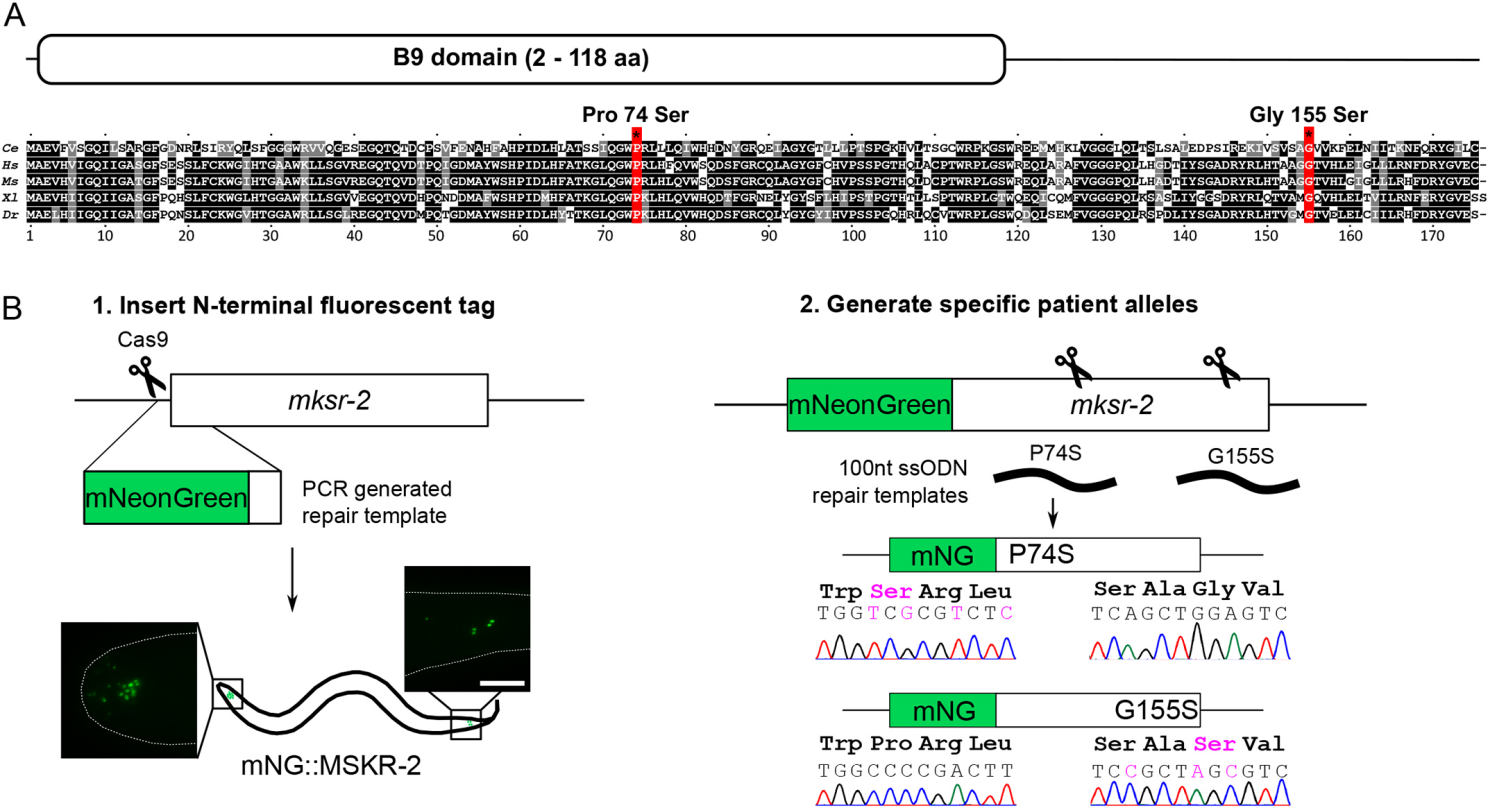
**CRISPR-Cas9 engineering of Joubert** **syndrome-associated P74S and G155S into endogenous *C. elegans mksr-2* gene.** (A) Protein alignment of B9D2 sequences from *C. elegans* (Q9N423), *Homo* *sapiens* (Q9BPU9), *Mus* *musculus* (Q3UK10), *Xenopus* *laevis* (Q6GN70) and *Danio* *rerio* (Q6DGZ1). UniprotKB accession numbers are indicated. B9D2 proteins contain a B9 domain (amino acids 2-118) and a conserved C-terminal tail, with no predicted domains. Two pathogenic Joubert syndrome variants (red), Pro74Ser and Gly155Ser, affect conserved residues. Protein sequences were aligned with Clustal Omega, and BoxShade v.3.21 was used to generate the figure. (B) CRISPR-Cas9 genome editing was used to introduce a fluorescent mNeonGreen (mNG) tag to the N-terminus of the endogenous *mksr-2* gene. Scale bar: 10 µm. The mNG::MKSR-2-tagged allele was then used to engineer two pathogenic missense variants, P74S and G155S. Silent and missense engineered mutations are shown in magenta. ssODN, single-stranded oligonucleotide. Fig. S1 shows detailed information about the CRISPR approach.

Human B9D2 and the *C. elegans* MKSR-2 orthologue possess 63.4% amino acid (aa) similarity (100% length, BLAST e-value 3×10^−52^; Fig. 1A). The protein is composed of a B9 domain (aa 2-118) and a C-terminal region with no predicted domains. Given that the B9D2 protein is highly conserved, we reasoned that *C. elegans* would be an excellent organism to model pathogenic patient variants of this protein in a biological system. Specifically, we focused on two missense variants: Pro74Ser in the B9 domain and Gly155Ser in the C-terminal region (Fig. 1A). Both variants are reported to be pathogenic in a biallelic patient with JBTS (Bachmann-Gagescu et al., 2015). Patient variants were compared with a 279 bp probably null deletion, *mksr-2(tm2452)* [hereafter termed *mksr-2(Δ)*] (Bialas et al., 2009; Williams et al., 2008, 2011).

Before generating the orthologous patient alleles, we used CRISPR-Cas9 to knock in *C. elegans* optimised mNeonGreen (mNG) with a short (7aa) flexible linker (GTGGGGS) at the 5′ end of the *mksr-2* locus (Fig. 1B). We performed this step to enable us to determine the effect of the mutations on the TZ localisation of endogenous MKSR-2. This generated the *mksr-2(oq108)* allele, which we refer to as *mNG::mksr-2(+)*. Next, we engineered the P74S and G155S variants into the *mNG::mksr-2(+)* strain, generating *mksr-2(oq108,oq125)/mNG::mksr-2(P74S)* and *mksr-2(oq108,oq126)/mNG::mksr-2(G155S)*. We also generated non-tagged versions of the variant alleles, *mksr-2(oq137)*/P74S and *mksr-2(oq138)*/G155S, using a modified PCR-based detection protocol (Fig. S1).

### MKSR-2 distribution at the TZ is severely altered by the G155S mutation

**Fig. 2.**
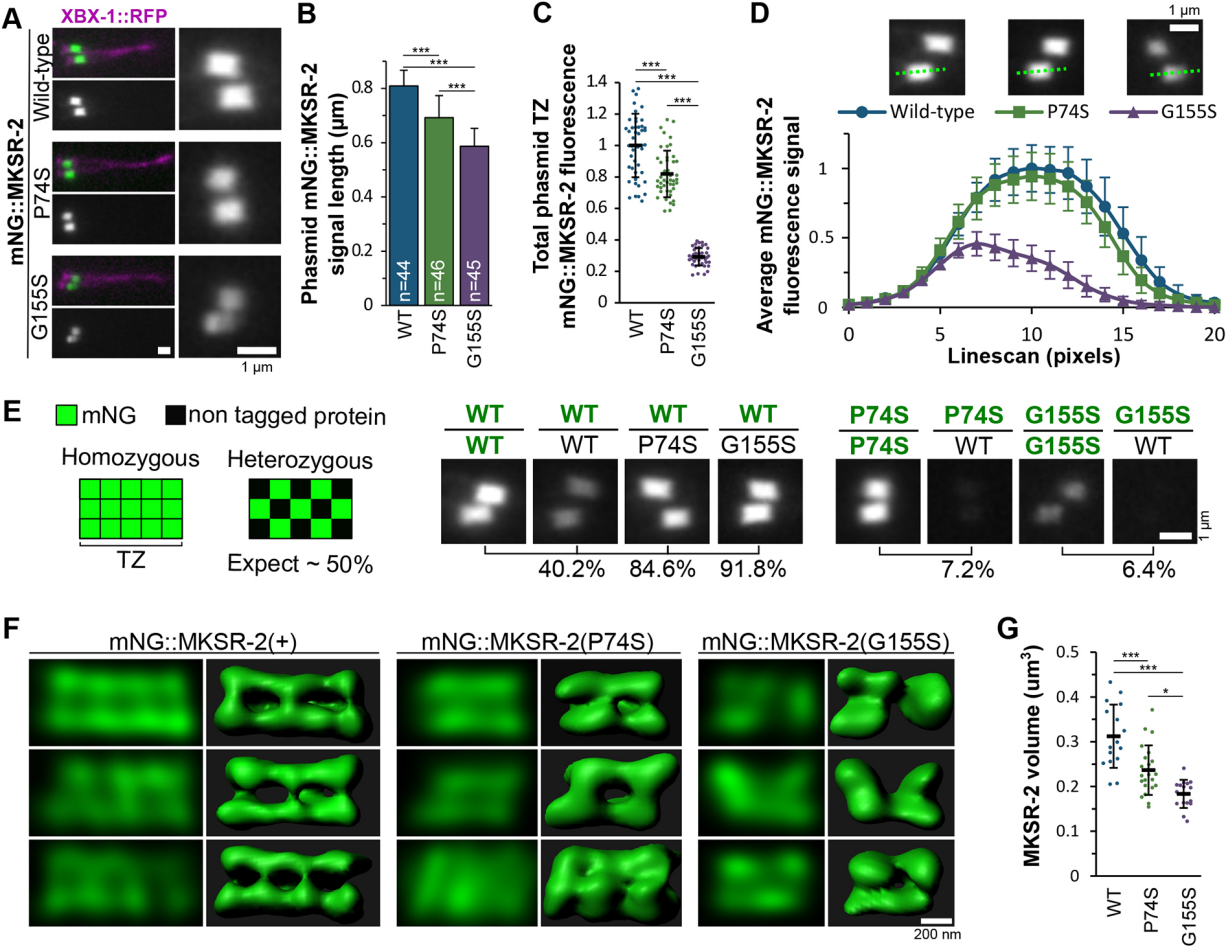
**P74S and G155S mutations differentially disrupt the recruitment and organisation of endogenous MKSR-2 at the transition zone (TZ).** (A) Representative images of one pair of phasmid cilia from worms expressing XBX-1::RFP (which marks the entire cilium) and endogenously tagged mNG::MKSR-2 (WT and variants). Magnified views of the TZs (side-on views) are shown in the right-hand panels. Anterior is to the left. (B) Quantification of mNG::MKSR-2 signal length by calculating the ‘full width at half max’ from a line scan of the phasmid TZ. Bar graph shows mean with s.d.; *n*, total number of TZs measured. Statistical significance was assessed by one-way ANOVA followed by Tukey's post hoc test (*P*-values: WT versus P74S, *P*=1.6×10^−12^; WT versus G155S, *P*=1.8×10^−15^; P74S versus G155S, *P*=8.6×10^−11^). (C) Quantification of the total fluorescence intensity of mNG::MKSR-2 at the TZ of phasmid cilia. Background fluorescence was subtracted, and values were normalised to WT. Black lines are the mean±s.d. Total TZ measured: WT (*n*=48), P74S (*n*=45), G155S (*n*=44). Statistical significance was assessed by one-way ANOVA followed by Tukey's post hoc test (*P*-values: WT versus P74S, *P*=1.3×10^−7^; WT versus G155S, *P*=2.2×10^−14^; P74S versus G155S, *P*=2.2×10^−14^). (D) Mean±s.d. fluorescence intensity from line scans (20 pixels=1.6 µm) of phasmid cilia showing the distribution of mNG::MKSR-2 along the TZ length. Green dotted lines show sample line scans. *n*=12 for each genotype. (E) Assessment of the efficiency of endogenous mNG::MKSR-2 recruitment to the phasmid TZ using strains heterozygous for the mNG::MKSR-2 alleles. Relative percentage fluorescence is indicated for each heterozygous genotype relative to the homozygous reference (100%). Green lettering indicates mNG-tagged allele of *mksr-2*. (F) Representative super-resolution confocal images of endogenous mNG::MKSR-2 in phasmid cilia. A single slice from the *z*-stack and corresponding 3D model are shown. All images are oriented with the proximal end of the TZ to the left. (G) Quantification of the volume of the mNG::MKSR-2 compartment at the TZ of phasmid cilia. Black lines are the mean±s.d. Total number of TZ measured: WT (*n*=16), P74S (*n*=22), G155S (*n*=16). Statistical significance was assessed by one-way ANOVA followed by Tukey's post hoc test (*P*-values: WT versus P74S, *P*=0.0003; WT versus G155S, *P*=6.3×10^−8^; P74S versus G155S, *P*=0.013). *P<0.05, ***P<0.001. Scale bars: 1 µm (A,D,E); 200 nm (F).

First, we investigated whether the P74S and G155S mutations affect the TZ localisation of MKSR-2. Analysis of the *mNG::mksr-2(+)* strain revealed that wild-type (WT) MKSR-2 was localised at the TZs of cilia in the head (labial and amphid) and tail (PQR and phasmid) of the worm (Fig. 2A; Fig. S2A). Previous reports of MKSR-2 localisation in *C. elegans*, using an overexpressed transgene, showed that MKSR-2::YFP localises at the TZ and in the more proximal periciliary region at the dendritic ending (Williams et al., 2008). Given that the endogenously expressed mNG::MKSR-2 showed no periciliary signal, we concluded that this previously reported pool of MKSR-2 was an overexpression artefact.

mNG::MKSR-2(P74S) and mNG::MKSR-2(G155S) were both observed at the TZ (Fig. 2A). However, the length of the TZ signal was 15-25% shorter than WT for both variants (Fig. 2B; Fig. S2B), and the overall levels of the mNG::MKSR-2 variants at the TZ were moderately (P74S) or severely (G155S) reduced (Fig. 2C). Line-scan analysis of the fluorescent signals revealed that mNG::MKSR-2(+) and mNG::MKSR-2(P74S) were homogeneously distributed along the TZ length; by contrast, mNG::MKSR-2(G155S) was asymmetrically distributed at the TZ, peaking at the proximal end (Fig. 2D).

Next, we investigated whether the variants affected the efficiency of endogenous MKSR-2 targeting to the TZ by examining their localisations when a WT copy of the gene was present. Genetic crosses were used to generate heterozygous worms (Fig. S3A). If the tagged protein were to be targeted to the TZ as efficiently as the untagged WT protein, then the fluorescent TZ signal should be half that observed in homozygous worms (Fig. 2E). Indeed, heterozygous mNG::MKSR-2(+)/+ worms showed ∼60% reduction in TZ fluorescence, indicating that mNG::MKSR-2(+) retained a near-WT ability to localise at the TZ (Fig. 2E; Fig. S3B). However, in worms heterozygous for mNG::MKSR-2(+), with either untagged MKSR-2(P74S) or MKSR-2(G155S), fluorescence was decreased by only 10-15%, indicating that the non-fluorescent mutant proteins were unable to compete with WT MKSR-2 for TZ incorporation (Fig. 2E; Fig. S3B). In support of this conclusion, the reverse experiment using mNG::MKSR-2(P74S)/+ and mNG::MKSR-2(G155S)/+ heterozygotes showed an almost complete loss of TZ localisation for the tagged variants when a non-fluorescent copy of WT MKSR-2 was present (Fig. 2E; Fig. S3B). These data indicate that both P74S and G155S decrease the efficiency of MKSR-2 recruitment to the TZ.

To assess the altered localisation of the MKSR-2 variants at the TZ, we used a scanning confocal-based super-resolution microscopy methodology (Lam et al., 2017). With confocal imaging using a reduced pinhole size and deconvolution, we obtained an optical resolution of ∼150 nm. Three-dimensional (3D) models of the phasmid TZ, in axial (longitudinal) view, were constructed from *z*-stacks. We found that mNG::MKSR-2(+) was excluded from the inner core of the TZ and displayed a periodic pattern at the outer (peripheral) region of the compartment, consisting of three or four peaks of axial signal (Fig. 2F). In some images, the signals appeared to wrap around the TZ, possibly as a spiral or ring (Fig. S4). This was somewhat similar to what we previously described using stimulated emission depletion (STED) microscopy for overexpressed reporters of other *C. elegans* MKS module proteins (Lambacher et al., 2016). mNG::MKSR-2(G155S) localisation was highly disrupted (Fig. 2F); typically, the protein was discontinuous along the TZ length, with 88% of analysed TZs (*n*=16) showing only two peaks. In addition, the ‘hollow TZ core’ was not always observed in the central plane of the images, and the 3D models showed that mNG::MKSR-2(G155S) occupied a reduced overall volume (Fig. 2G). The super-resolution imaging also provided evidence of subtle TZ distribution defects for mNG::MKSR-2(P74S), which displayed only two or three peaks of axial signal, a less pronounced spiral/ring pattern and a modestly reduced volume of occupation, although the ‘hollow core’ pattern was retained (Fig. 2F,G).

Overall, these data indicate that both P74S and G155S affect the TZ targeting and distribution of MKSR-2. Although both alleles are pathogenic, the G155S variant has a more severe effect on MKSR-2 organisation at the TZ.

### P74S and G155S mutations in *mksr-2* differentially disrupt ciliary structure, positioning and function

In *C. elegans*, the MKS and NPHP modules interact genetically to regulate cilium formation and function (Williams et al., 2008, 2011). For example, whereas ciliogenesis is mostly unaffected in single null mutants of *mksr-2* and *nphp-4*, severe ciliary defects and sensory behaviours are observed in *mksr-2; nphp-4* double null mutants (Williams et al., 2008). Therefore, to investigate the phenotypic effects of the *mksr-2* patient alleles, we assessed sensory cilia in both WT/*nphp-4(+)* and *nphp-4(Δ)* genetic backgrounds.

**Fig. 3.**
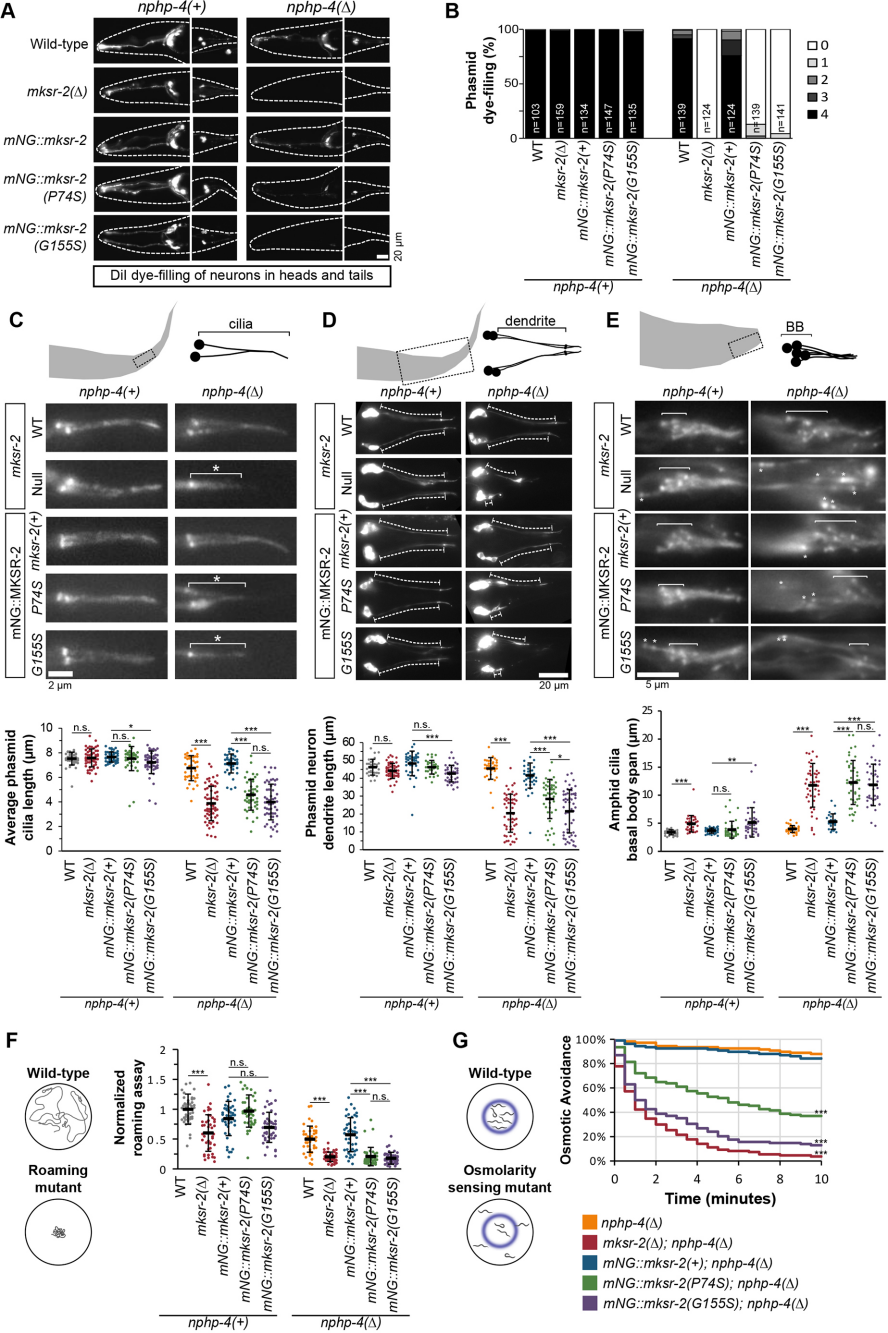
**P74S and G155S mutations in** ***mksr-2*** **differentially disrupt cilium structure and function.** (A) Representative images of DiI staining in head and tail ciliated sensory neurons of various *mksr-2* alleles in either *nphp-4(+)* or *nphp-4(Δ)* genetic backgrounds. Dashed line indicates the worm body. Anterior is to the left. (B) Histogram showing the frequency of worms with DiO uptake in zero to four phasmid neurons. Data were combined from three independent experiments. *n*, number of worms. (C) Assessment of phasmid cilium length using an XBX-1::tdTomato reporter. White asterisks denote short cilia. Black lines are the mean±s.d. Total number of cilia: WT (*n*=41), *mksr-2* (*n*=55), *mNG::mksr-2(+)* (*n*=48), *mNG::mksr-2(P74S)* (*n*=41), *mNG::mksr-2(G155S)* (*n*=50), *nphp-4* (*n*=39), *nphp-4;mksr-2* (*n*=63), *nphp-4;mNG::mksr-2(+)* (*n*=44), *nphp-4;mNG::mksr-2(P74S)* (*n*=47), *nphp-4;mNG::mksr-2(G155S)* (*n*=53). Statistical significance was assessed by one-way ANOVA followed by Tukey's post hoc test [*P*-values: *mNG::mksr-2(+)* versus *mNG::mksr-2(G155S)*, *P*=0.032; *nphp-4* versus *nphp-4;mksr-2*, *P*=9.1×10^−15^; *nphp-4;mNG::mksr-2(+)* versus *nphp-4;mNG::mksr-2(P74S)*, *P*=8.9×10^−15^; *nphp-4;mNG::mksr-2(+)* versus *nphp-4;mNG::mksr-2(G155S)*, *P*=9.1×10^−15^]. (D) Assessment of dendrite length using an XBX-1::tdTomato reporter. Dashed lines denote the dendrites. Anterior is to the left. Black lines are the mean±s.d. Total number of dendrites: WT (*n*=31), *mksr-2* (*n*=53), *mNG::mksr-2(+)* (*n*=39), *mNG::mksr-2(P74S)* (*n*=32), *mNG::mksr-2(G155S)* (*n*=43), *nphp-4* (*n*=34), *nphp-4;mksr-2* (*n*=53), *nphp-4;mNG::mksr-2(+)* (*n*=45), *nphp-4;mNG::mksr-2(P74S)* (*n*=44), *nphp-4;mNG::mksr-2(G155S)* (*n*=58). Statistical significance was assessed by one-way ANOVA followed by Tukey's post hoc test [*P*-values: *mNG::mksr-2(+)* versus *mNG::mksr-2(G155S)*, *P*=4.7×10^−6^; *nphp-4* versus *nphp-4;mksr-2*, *P*=2.4×10^−14^; *nphp-4;mNG::mksr-2(+)* versus *nphp-4;mNG::mksr-2(P74S)*, *P*=2.6×10^−8^; *nphp-4;mNG::mksr-2(+)* versus *nphp-4;mNG::mksr-2(G155S)*, *P*=2.3×10^−14^; *nphp-4;mNG::mksr-2(P74S)* versus *nphp-4;mNG::mksr-2(G155S)*, *P*=0.012]. (E) Assessment of amphid cilia clustering using the XBX-1::tdTomato reporter. White asterisks indicate amphid neuron basal bodies (and cilia) that are mispositioned, proximal to the main cluster. Anterior is to the right. Black lines are the mean±s.d. Total number of worms: WT (*n*=37), *mksr-2* (*n*=34), *mNG::mksr-2(+)* (*n*=41), *mNG::mksr-2(P74S)* (*n*=43), *mNG::mksr-2(G155S)* (*n*=40), *nphp-4* (*n*=40), *nphp-4;mksr-2* (*n*=44), *nphp-4;mNG::mksr-2(+)* (*n*=26), *nphp-4;mNG::mksr-2(P74S)* (*n*=44), *nphp-4;mNG::mksr-2(G155S)* (*n*=37). Statistical significance was assessed by Kruskal–Wallis test followed by Dunn's post hoc test [*P*-values: WT versus *mksr-2*, *P*=4.9×10^−8^; *mNG::mksr-2(+)* versus *mNG::mksr-2(G155S)*, *P*=0.0012; *nphp-4* versus *nphp-4;mksr-2*, *P*=6.7×10^−16^; *nphp-4;mNG::mksr-2(+)* versus *nphp-4;mNG::mksr-2(P74S)*, *P*=1.2×10^−8^; *nphp-4;mNG::mksr-2(+)* versus *nphp-4;mNG::mksr-2(G155S)*, *P*=1.7×10^−7^]. (F) Assessment of worm roaming behaviour normalised to WT. Data were pooled from three independent experiments. Black lines are the mean±s.d. Total number of worms: WT (*n*=46), *mksr-2* (*n*=44), *mNG::mksr- 2(+)* (*n*=44), *mNG::mksr-2(P74S)* (*n*=43), *mNG::mksr-2(G155S)* (*n*=44), *nphp-4* (*n*=44), *nphp-4;mksr-2* (*n*=43), *nphp-4;mNG::mksr-2(+)* (*n*=44), *nphp-4;mNG::mksr-2(P74S)* (*n*=43), *nphp-4;mNG::mksr-2(G155S)* (*n*=46). Statistical significance was assessed by one-way ANOVA followed by Tukey's post hoc test [*P*-values: WT versus *mksr-2*, *P*=5.4×10^−10^; *nphp-4* versus *nphp-4;mksr-2*, *P*=1.8×10^−11^; *nphp-4;mNG::mksr-2(+)* versus *nphp-4;mNG::mksr-2(P74S)*, *P*=3.1×10^−14^; *nphp-4;mNG::mksr-2(+)* versus *nphp-4;mNG::mksr-2(G155S)*, *P*=3.1×10^−14^]. (G) Assessment of osmotic avoidance behaviour in a 10-min assay. For each genotype, *n*=18. Statistical significance was assessed by Kruskal–Wallis test followed by Dunn's post hoc test at 10 min [*P*-values: *nphp-4* versus *nphp-4;mksr-2*, *P*=9.8×10^−11^; *nphp-4;mNG::mksr-2(+)* versus *nphp-4;mNG::mksr-2(P74S)*, *P*=0.0007; *nphp-4;mNG::mksr-2(+)* versus *nphp-4;mNG::mksr-2(G155S)*, *P*=3.8×10^−7^]. *P<0.05, ***P<0.001; n.s., not significant. Scale bars: 2 µm (C); 5 µm (E); 20 nm (A,D).

We used a lipophilic dye uptake assay to examine the integrity of the cilium of amphid (head) and four phasmid (tail) sensory neurons indirectly (Inglis et al., 2007; Starich et al., 1995). In the *nphp-4(+)* genetic background, none of the *mksr-2* alleles affected dye uptake (Fig. 3A,B). However, in the *nphp-4(Δ)* background, the *mNG::mksr-2(P74S)* and *mNG::mksr-2(G155S)* alleles both caused very strong dye-filling defects, which were almost as severe as that of the *mksr-2(Δ)* null allele (Fig. 3A,B). The *mNG::mksr-2(+)* control allele caused a slight dye-filling defect, indicating that the mNG tag negatively affected *mksr-2* function to some extent.

Next, we used an intraflagellar transport (IFT)-dynein light intermediate chain reporter (XBX-1::tdTomato) to visualise cilia directly (Williams et al., 2011). In the *nphp-4(+)* background, phasmid cilium length was decreased only modestly in *mNG::mksr-2(G155S)* (Fig. 3C). In the *nphp-4(Δ)* background, a 30-40% reduction in cilium length was associated with the *mNG::mksr-2(P74S)*, *mNG::mksr-2(G155S)* and *mksr-2(Δ)* alleles, compared with the *mksr-2(+)* control (Fig. 3C). Using the XBX-1::tdTomato reporter, we also assessed cilium location, because loss of MKS module genes in an *nphp-4(Δ)* background disrupts the positioning of some cilia on account of abnormally short dendrites (Williams et al., 2008, 2011). For the phasmids, we measured dendrite length; for the amphids, we determined the spatial arrangement of their cilia by measuring the degree of basal body (BB) clustering. In the *nphp-4(+)* background, subtle defects in cilium positioning were observed in *mksr-2(Δ)* and *mNG::mksr-2(G155S)* (Fig. 3D,E). In the *nphp-4(Δ)* background, *mksr-2(Δ)*, *mNG::mksr-2(G155S)* and *mNG::mksr-2(P74S)* alleles showed a reduction in phasmid dendrite length (Fig. 3D) and amphid neuron BBs that were less tightly clustered (Fig. 3E).

To determine whether the *mksr-2* patient alleles affected the function of cilia, we assessed two cilia-dependent sensory behaviours: foraging and osmotic avoidance. WT *C. elegans* display a food foraging behaviour, which can be quantified via a roaming assay (Fig. 3F). In the *nphp-4(+)* background, the *mksr-2(Δ)* allele caused a significant reduction in roaming behaviour, whereas the *mNG::mksr-2(P74S)* and *mNG::mksr-2(G155S)* alleles did not (*P*-values of 0.214 and 0.087, respectively) (Fig. 3F). In the *nphp-4(Δ)* background, the *mksr-2(Δ)*, *mNG::mksr-2(G155S)* and *mNG::mksr-2(P74S)* alleles all caused a strong roaming defect (Fig. 3F). In the osmotic avoidance assay, worms are assessed for their ability to avoid crossing a barrier of high osmolarity (Fig. 3G). In the *nphp-4(+)* background, none of the *mksr-2* alleles had a significant osmotic avoidance defect (data not shown). In the *nphp-4(Δ)* genetic background, the *mNG::mksr-2(P74S)* allele had a moderate defect, whereas the *mNG::mksr-2(G155S)* and *mksr-2(Δ)* alleles caused a strong reduction in osmotic sensing (Fig. 3G).

### P74S and G155S mutations in *mksr-2* disrupt ciliary and TZ ultrastructure

**Fig. 4.**
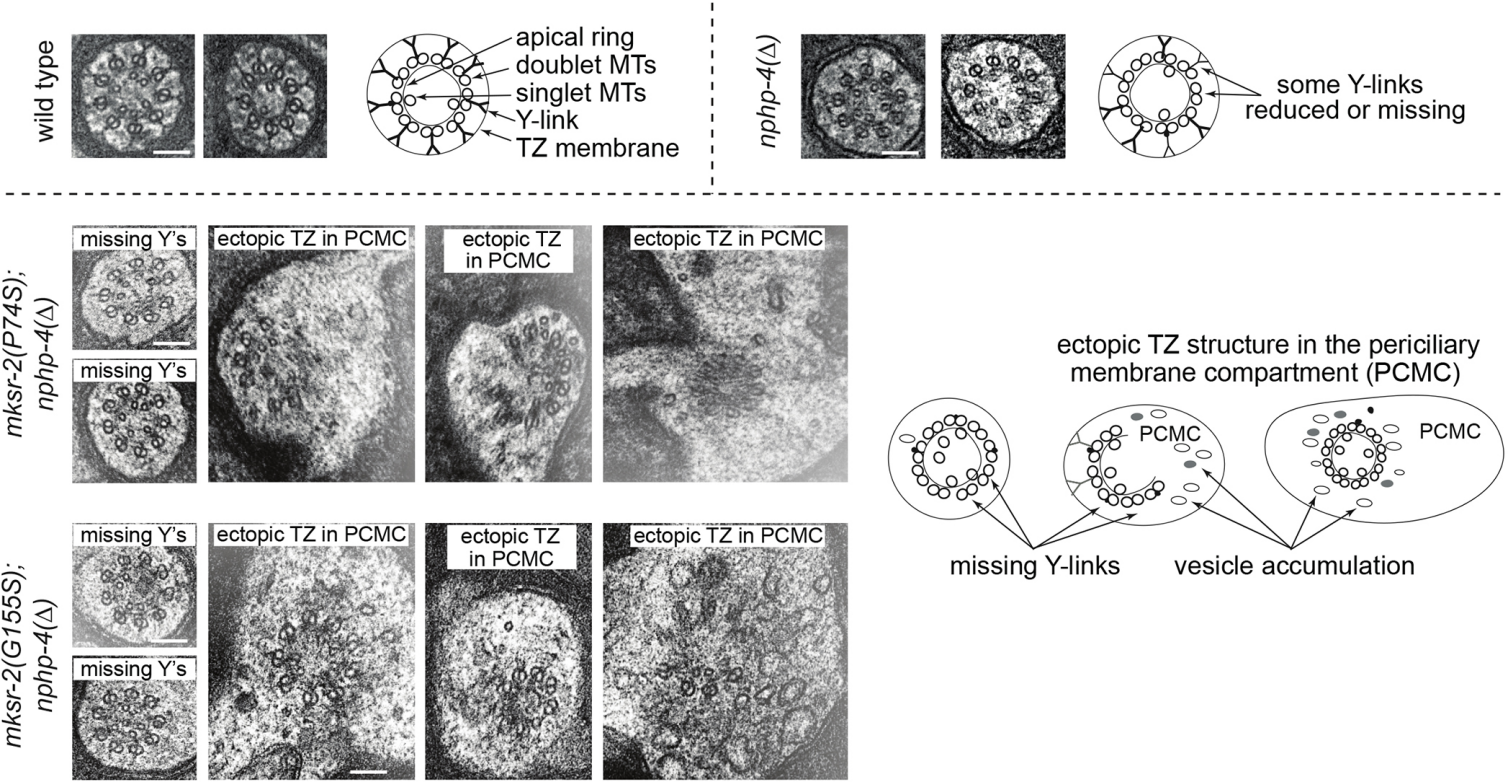
**Ultrastructure of the transition zone is disrupted by P74S and G155S mutations in *mksr-2*.** Transmission electron micrographs of amphid channel cilia transition zones in cross-section (radial orientation) in *mksr-2(P74S); nphp-4(Δ)* and *mksr-2(G155S); nphp-4(Δ)* double mutants. Note that the WT and *nphp-4(Δ)* control images are adapted from Lambacher et al. (2016). These images are not published under the terms of the CC-BY license of this article. For permission to reuse, please see Lambacher et al. (2016). PCMC, periciliary membrane compartment; Y's, Y-linkers. Scale bars: 100 nm.

We performed transmission electron microscopy to address the effects of the *mksr-2* P74S and G155S mutations on TZ ultrastructure. As with general cilia structure phenotypes described above, defects in TZ structure and positioning are typically observed only in worms with null mutations in *nphp-4* and an MKS module gene (e.g. *mksr-1*), and not in most corresponding single mutants, with the exception of *mks-5* and *cep-290* (Jensen et al., 2015; Li et al., 2016; Williams et al., 2011). Thus, we analysed TZ ultrastructure in *mksr-2(P74S);nphp-4(Δ)* and *mksr-2(G155S);nphp-4(Δ)* double mutants. Specifically, we analysed serial thin sections of the bilateral amphid pore, each of which possesses 10 channel cilia extending from the distal dendrites of eight amphid neurons (Doroquez et al., 2014; Perkins et al., 1986). These cilia consist of a degenerate basal body at the distal-most end of the dendrite, docked at the membrane of the periciliary membrane compartment (PCMC), followed by an ∼0.8-μm-long TZ, with Y-links connecting the nine closely tethered outer doublet microtubules (MTs) to the ciliary membrane (Fig. 4). Following the TZ is the ∼3-μm-long middle segment, which consists of nine doublet MTs. The amphid channel cilia end with a ∼3-μm-long distal segment, comprising nine singlet MTs owing to outer doublet B-tubule termination at the end of the middle segment. Amphid channel cilia also contain varying numbers of inner singlet MTs.

The ultrastructure of *nphp-4(Δ)* (control) amphid channel cilia resembled that of WT worms, except for a slight truncation of one to three cilia (Fig. S5) (Jauregui et al., 2008). *nphp-4(Δ)* worms also showed a reduction in the density of some Y-linkers in the TZ and, in a few cases, ectopic TZ structure was observed in the cytoplasm of the PCMC (Fig. 4; Fig. S5, Table S1) (Lambacher et al., 2016). By contrast, the *mksr-2(P74S);nphp-4(Δ)* and *mksr-2(G155S);nphp-4(Δ)* double mutants showed more severe ciliary and TZ ultrastructural defects. Five to eight amphid channel cilia were moderately or severely truncated, and, in some cases, cilia were mispositioned in more proximal positions or extended at oblique angles to the other axonemes (Fig. 4; Fig. S5, Table S1). Compared with the *nphp-4(Δ)* control, double mutants showed no alteration in the number of microtubule singlets and doublets, although it was interesting to note that the frequency of axonemes with unzipped B-tubules might be increased in *mksr-2(P74S);nphp-4(Δ)* worms and reduced in *mksr-2(G155S);nphp-4(Δ)* worms (Table S1). Both double mutants showed a similar degree of TZ disruption, with Y-links typically reduced in electron density or absent (Fig. 4). Also, in the majority of cases, the double mutants displayed ectopic TZ structure within the cytoplasm of the PCMC. Furthermore, the double mutant PCMCs often displayed a large accumulation of vesicles (Fig. 4; Table S1). Thus, the P74S and G155S mutations in *mksr-2* exacerbated the ciliary and TZ defects associated with *nphp-4* loss. It was notable, however, that despite distinctions between *mksr-2(P74S);nphp-4(Δ)* and *mksr-2(G155S);nphp-4(Δ)* in their dye-filling and chemosensing ability (Fig. 3), we were unable to identify specific differences in their respective ciliary ultrastructural phenotypes.

### Ciliary membrane gating of RPI-2 and TRAM-1a is disrupted by the G155S, but not P74S, mutation in *mksr-2*

**Fig. 5.**
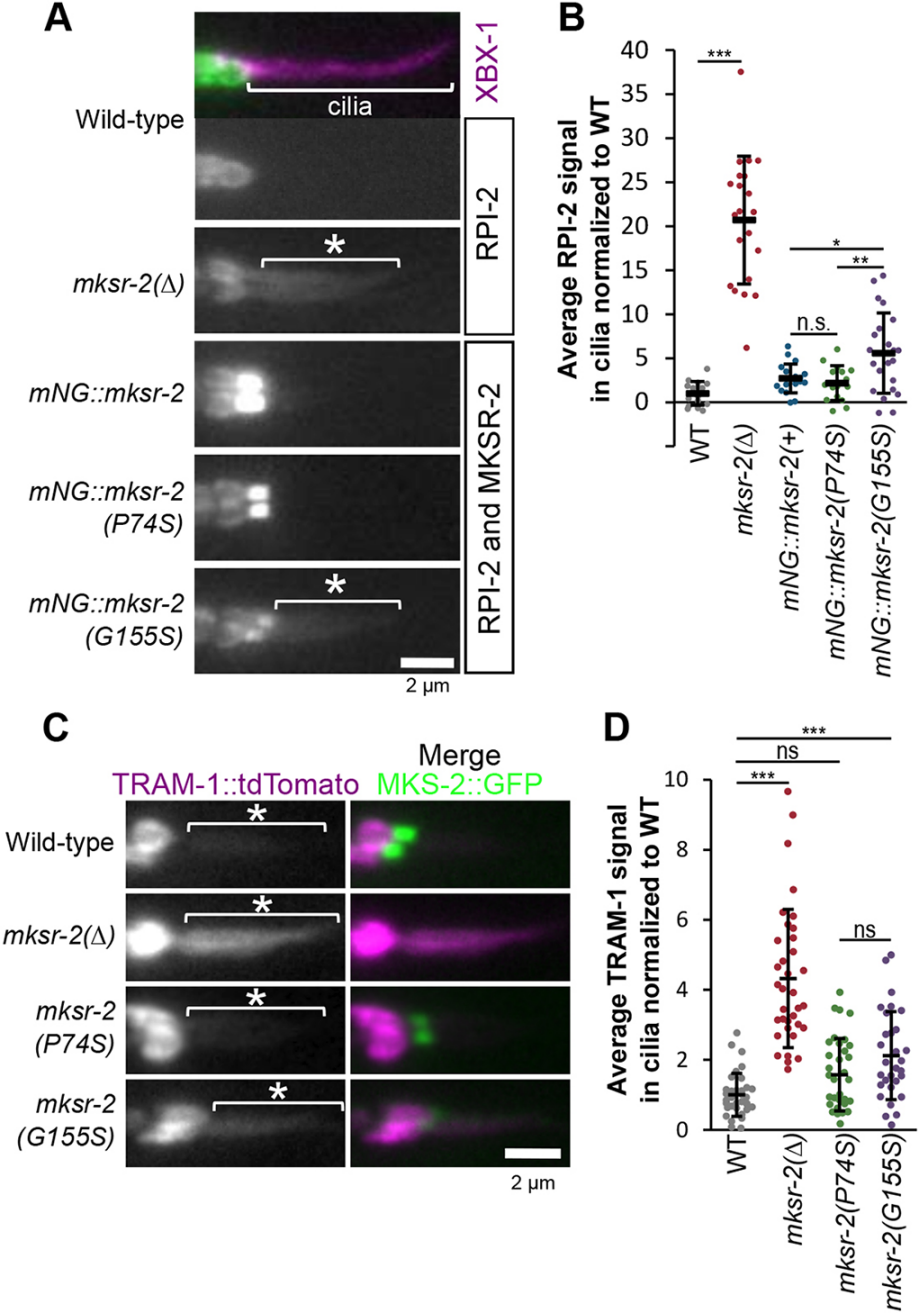
**G155S mutation in** ***mksr-2*** **causes the periciliary membrane proteins RPI-2 and TRAM-1a to leak into cilia.** (A) Representative images of phasmid cilia expressing the ciliary marker XBX-1::tdTomato and a reporter of the periciliary membrane protein, RPI-2::eGFP. White asterisks indicate RPI-2 signal in cilia. Note that XBX-1 signals are shown only in the top image. Anterior is to the left. (B) Quantification of RPI-2::GFP signal in phasmid cilia. Background fluorescence was subtracted, and values were normalised to WT. Black lines are the mean±s.d. Total number of cilia: WT (*n*=17), *mksr-2* (*n*=21), *mNG::mksr-2(+)* (*n*=19), *mNG::mksr-2(P74S)* (*n*=16), *mNG::mksr-2(G155S)* (*n*=22). Statistical significance was assessed by one-way ANOVA followed by Tukey's post hoc test [*P*-values: WT versus *mksr-2*, *P*=4.6×10^−13^; *mNG::mksr-2(+)* versus *mNG::mksr-2(G155S)*, *P*=0.015; *mNG::mksr-2(P74S)* versus *mNG::mksr-2(G155S)*, *P*=0.005]. (C) Representative images of phasmid cilia expressing extrachromosomal TRAM-1::tdTomato and MKS-2::GFP. White asterisks indicate TRAM-1 signal in cilia. Anterior is to the left. (D) Quantification of TRAM-1::tdTomato in the cilia. Background fluorescence was subtracted, and values were normalised to WT. Black lines are the mean±s.d. Total number of cilia: WT (*n*=35), *mksr-2* (*n*=35), *mksr-2(P74S)* (*n*=34), *mksr-2(G155S)* (*n*=30). Statistical significance was assessed by the Kruskal–Wallis test followed by Dunn's post hoc test (*P*-values: WT versus *mksr-2*, *P*=9.55×10^−15^; WT versus P74S, *P*=0.064; WT versus G155S, *P*=0.0006; P74S versus G155S, *P*=0.103). *P<0.05, **P<0.01, ***P<0.001; n.s., not significant. Scale bars: 2 µm.

Next, we investigated whether the P74S and G155S mutations in *mksr-2* affected TZ gating of membrane proteins. In WT worms, a fluorescent lipidated RPI-2 (orthologue of human RP2 activator of ARL3 GTPase) reporter is enriched at the periciliary membrane and excluded from cilia (Williams et al., 2011). Consistent with previous findings (Williams et al., 2011), we found that RPI-2::GFP leaked into the cilia of *mksr-2(Δ)* mutants (Fig. 5A,B). The RPI-2 reporter also leaked into the cilia of *mNG::mksr-2(G155S)* worms, albeit not to the same extent as in the null allele (Fig. 5A,B). By contrast, the *mNG::mksr-2(P74S)* allele did not affect RPI-2 localisation, which remained excluded from cilia (Fig. 5A,B). Note that mNG::MKSR-2 and RPI-2::GFP fluorescence could not be distinguished in this experiment. Given that mNG::MKSR-2 was never observed in the WT cilium distal to the TZ (Fig. 2A), the green fluorescent signal observed in *mNG::mksr-2(G155S)*-tagged cilia was assumed to be RPI-2::GFP. To probe TZ gating further, we examined a transmembrane TRAM-1a reporter in untagged *mksr-2(P74S)* and *mksr-2(G155S)* alleles (Fig. 5C,D). Like RPI-2::GFP, the TRAM-1a::tdTomato reporter also localised ectopically to the cilia of *mksr-2(G155S)*, but not *mksr-2(P74S)*, worms. Thus, the G155S mutation in MKSR-2 somewhat disrupted ciliary membrane gating of RPI-2 and TRAM-1a, whereas this was not the case for the P74S mutation.

### TZ distribution of MKS-2 (core MKS module) is more severely affected than MKS-6 and JBTS-14 (peripheral MKS module) in worms with *mksr-2* patient mutations

**Fig. 6.**
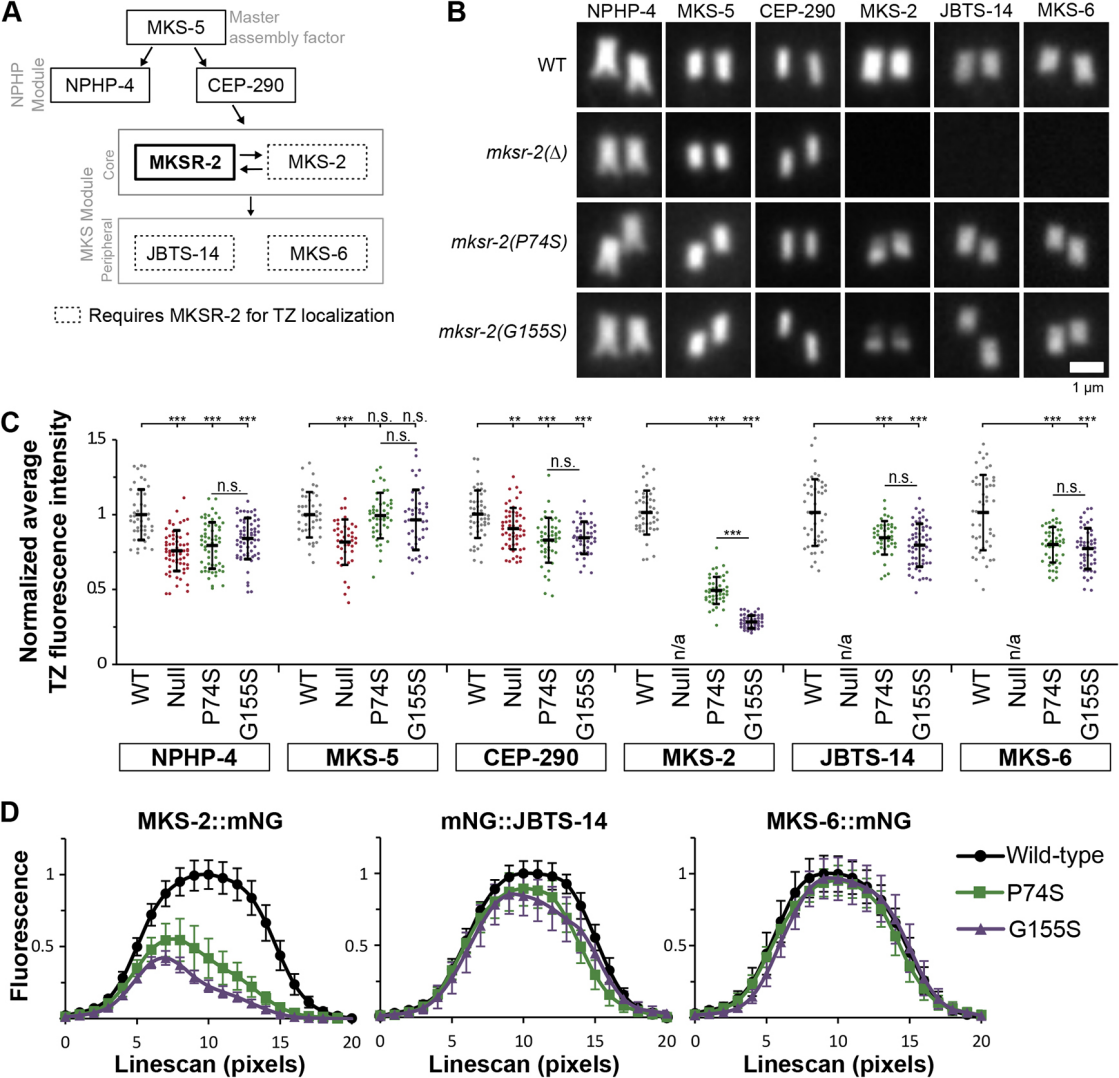
**P74S and G155S mutations in** ***mksr-2*** **disrupt MKS module protein distribution at the transition zone to different extents.** (A) Schematic diagram showing the position and role of MKSR-2 within the hierarchical model of NPHP and MKS module assembly at the TZ. MKSR-2 is a component of the core MKS module. Note that this is a simplified model, with only six MKS/NPHP proteins shown. A more complete model can be found in Li et al. (2016). This image has been reproduced from Li et al. (2016) under the terms of the CC-BY 4.0 license. (B) Representative images of phasmid neuron TZs from worms expressing endogenous mNG::NPHP-4, mNG::MKS-5, CEP-290::mNG, MKS-2::mNG, mNG::JBTS-14 and MKS-6::mNG. Proximal is to the bottom. Scale bar: 1 µm. (C) Quantification of the total fluorescence intensity of mNG at the TZ of phasmid cilia. Background fluorescence was subtracted, and values were normalised to WT. Black lines are the mean±s.d. *n* is the number of phasmid TZ pairs measured: NPHP-4 [WT (*n*=42), null (*n*=66), P74S (*n*=58), G155S (*n*=66)], MKS-5 [WT (*n*=42), null (*n*=40), P74S (*n*=46), G155S (*n*=42)], CEP-290 [WT (*n*=48), null (*n*=55), P74S (*n*=47), G155S (*n*=45)], MKS-2 [WT (*n*=44), null (*n*=53), P74S (*n*=50), G155S (*n*=52)], JBTS-14 [WT (*n*=45), null (*n*=50), P74S (*n*=45), G155S (*n*=59)] and MKS-6 [WT (*n*=47), null (*n*=53), P74S (*n*=48), G155S (*n*=49)]. Statistical significance was assessed by one-way ANOVA followed by Tukey's post hoc test [*P*-values: NPHP-4 (WT versus null, *P*=4.7×10^−14^; WT versus P74S, *P*=3.2×10^−10^; WT versus G155S, *P*=7.2×10^−7^), MKS-5 (WT versus null, *P*=8×10^−6^), CEP-290 (WT versus null, *P*=0.004; WT versus P74S, *P*=5.1×10^−8^; WT versus G155S, *P*=1.1×10^−6^), MKS-2 (WT versus P74S, *P*=2.5×10^−14^; WT versus G155S, *P*=2.5×10^−14^; P74S versus G155S, *P*=2.4×10^−14^), JBTS-14 (WT versus P74S, *P*=7.9×10^−6^; WT versus G155S, *P*=1.1×10^−9^) and MKS-6 (WT versus P74S, *P*=8.6×10^−8^; WT versus G155S, *P*=2×10^−9^)]. Fluorescence levels of MKS-2, JBTS-14 and MKS-6 in the *mksr-2* null mutant background were not quantified because there was no TZ signal observed. (D) Average fluorescence intensity from line scans (20 pixels=1.6 µm) of phasmid cilia showing the distribution of MKS-2, JBTS-14 and MKS-6 along the TZ length. Values were normalised to WT. *n*=12 for each genotype. Data represent the mean±s.d. **P<0.01, ***P<0.001; n.s., not significant.

MKSR-2 resides within the B9 complex (with MKS-1/MKS1 and MKSR-1/B9D1), which is part of the MKS module core required for the TZ recruitment of other core (MKS-2/TMEM216) and peripheral (JBST-14/TMEM237 and MKS-6/CC2D2A) MKS module proteins. The MKS module core is not required for the TZ recruitment of the NPHP or CEP290 modules, nor for the master assembly factor MKS-5 (RPGRIP1L orthologue) (Fig. 6A) (Huang et al., 2011; Jensen et al., 2015; Lambacher et al., 2016; Li et al., 2016; Williams et al., 2011). To examine the localisation of TZ module reporters expressed at endogenous levels, we used CRISPR-Cas9 to knock in mNG at the *mks-5*, *nphp-4*, *cep-290*, *mks-2*, *jbts-14* and *mks-6* loci (Fig. S6A).

Initially, we examined endogenous reporter localisations in *mksr-2(Δ)* worms. Consistent with previous reports (Huang et al., 2011; Jensen et al., 2015; Li et al., 2016; Williams et al., 2011), we found that the *mksr-2(Δ)* null mutant had lost TZ localisation of mNG-tagged MKS-2, JBTS-14 and MKS-6, but retained TZ localisation of NPHP-4, CEP-290 and MKS-5 (Fig. 6B; Fig. S6D). Interestingly, our quantitative measurements revealed that the *mksr-2(Δ)* mutation also caused a slight decrease in the TZ levels of endogenous mNG::NPHP-4, mNG::MKS-5 and CEP-290::mNG (Fig. 6C). Thus, although the MKS core module is not thought to be involved in the TZ recruitment of CEP-290, MKS-5 or the NPHP module (Jensen et al., 2015; Li et al., 2016; Schouteden et al., 2015; Williams et al., 2011), our data with endogenous reporters suggest that MKSR-2 is required for fully efficient TZ recruitment or retention of these proteins. Two additional notable distinctions from reported data were also observed. First, the endogenous mNG::MKS-5 reporter localised as a continuous, somewhat shorter, signal at the TZ of the *mksr-2(Δ)* mutant (Fig. S6B), rather than an elongated signal as was previously reported for an overexpressed MKS-5::tdTomato reporter (Jensen et al., 2015). Indeed, a continuous mNG::MKS-5 signal was also found at the TZ region of *nphp-4*, *mks-2* and *nphp-4; mks-2* mutants (Fig. S6C), unlike previous findings with MKS-5::tdTomato (Jensen et al., 2015). Second, the endogenous mNG::MKS-2 reporter did not localise ectopically along the length of the *mksr-2(Δ)* mutant ciliary membrane as reported for an overexpressed MKS-2::GFP reporter (Huang et al., 2011). We conclude that these distinctions relate to the endogenous versus overexpressed nature of the reporters.

In contrast to the *mksr-2(Δ)* null allele, the endogenous mNG-tagged MKS-2, JBTS-14 and MKS-6 reporters were recruited to the ciliary TZs in *mksr-2(P74S)* and *mksr-2(G155S)* worms (Fig. 6B). However, reporter fluorescence levels at the TZs were affected in these worms, albeit to different extents. The mNG::JBST-14 and MKS-6::mNG signals were reduced by ∼20%, whereas the effect on MKS-2::mNG was more dramatic, showing a 50-75% reduction (most severe in G155S worms) (Fig. 6C). The P74S and G155S mutations also caused a small reduction in the TZ levels of NPHP-4::mNG and mNG::CEP-290 levels, as was seen in the null allele; although, unlike the null, the P74S and G155S mutations did not disrupt the TZ levels of mNG::MKS-5 (Fig. 6C; Fig. S6B). Interestingly, in the *mksr-2* variants, the MKS-2::mNG signal was not evenly distributed at the TZ; instead, fluorescence clearly peaked at the proximal end (Fig. 6D). By contrast, the patient variants did not cause a dramatic proximal shift in the TZ localisation of JBTS-14 and MKS-6, although mNG::JBTS-14 showed a slight proximal shift in *mksr-2(G155S)* worms (Fig. 6D). Finally, compared with WT worms, we found no effect on the TZ localisation of MKS-2::mNG in worms heterozygous for the P74S, G155S or null alleles (Fig. S6E,F).

Together, these data show that recessive P74S and G155S mutations in *mksr-2* differentially disrupt the TZ localisations of core and peripheral MKS module proteins. Notably, the TZ localisation of MKS-2 (core MKS module) is much more strongly affected than JBTS-14 and MKS-6 (peripheral MKS module), indicating a very close functional association between the B9 complex and MKS-2 (TMEM216 orthologue).

### Using *C. elegans* genetic crosses to mimic ‘carrier’ and ‘pathogenic’ genotypes

**Fig. 7.**
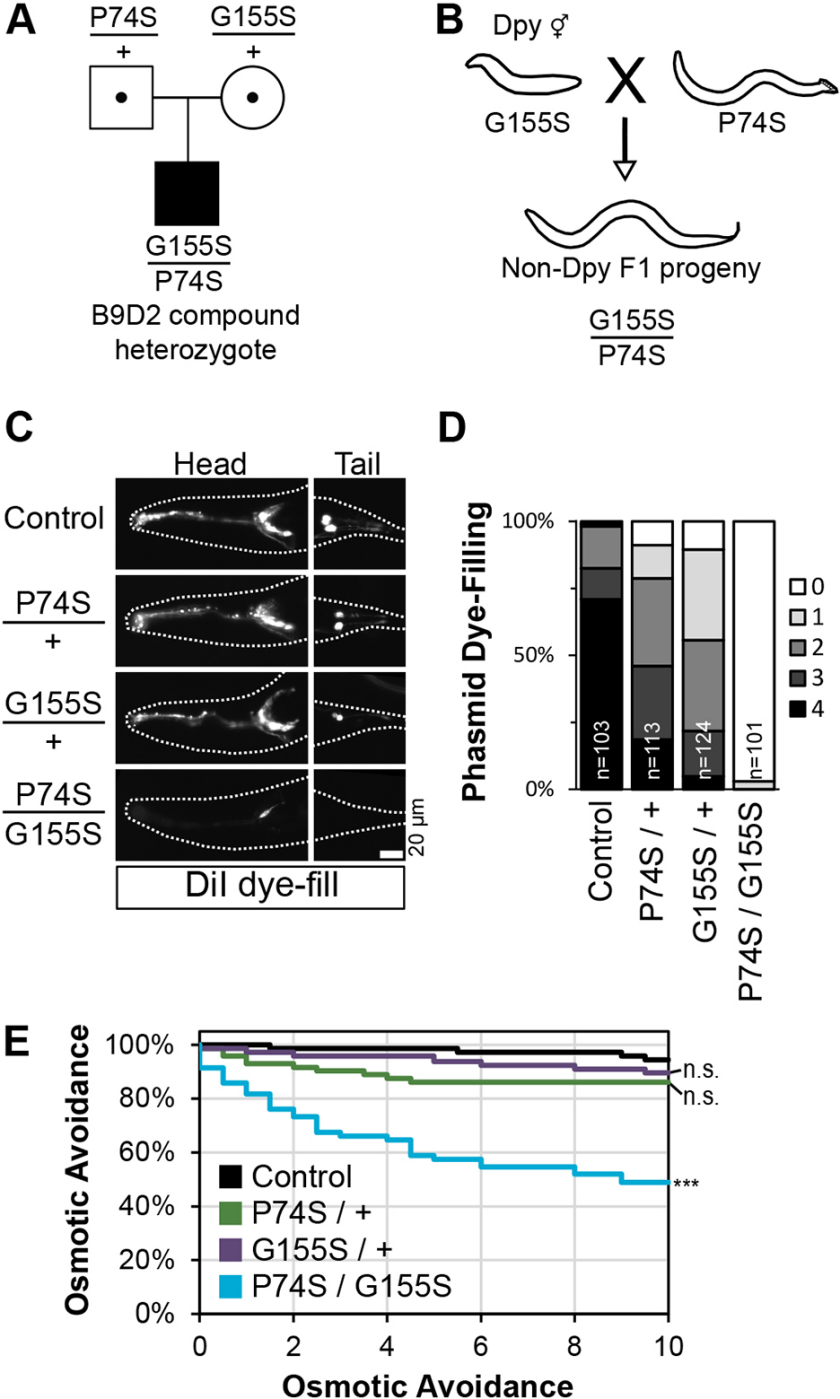
**Phenotypic interpretation of *C. elegans* compound heterozygous G155S/P74S worms that mimic the biallelic patient genotype.** (A) Pedigree of a patient with JBTS described by Bachmann-Gagescu et al. (2015). The parents are heterozygous carriers of P74S or G155S. (B) Genetic cross illustrating how compound heterozygous F1 progeny are generated in *C. elegans*. A recessive *dpy-5* allele ensures that analysis is performed only with outcrossed progeny. Full genotypes of genetic crosses used to generate heterozygous F1 worms are shown in Fig. S7. (C) Representative dye uptake images in *mksr-2* heterozygotes that are homozygous for the *nphp-4* deletion allele. Anterior is to the left. Scale bar: 20 µm. (D) Quantification of phasmid dye uptake. Histogram shows the frequency of worms with dye uptake in zero to four phasmid neurons. Data are from three pooled independent experiments. *n*, number of worms. (E) Assessment of osmotic avoidance behaviour in a 10-min assay. *n*=12 from three independent experiments. Statistical significance was assessed by the Kruskal–Wallis test followed by Dunn's post hoc test at the final 10-min time point (*P*-values: control versus P74S/G155S, 6.7×10^−6^). n.s., not significant.

Our analysis thus far has mostly examined the MKSR-2 variants in a homozygous state. However, this does not reflect the compound heterozygous genotype of the JBTS patient who inherited the two distinct *B9D2* variant alleles from their carrier parents (Fig. 7A) (Bachmann-Gagescu et al., 2015). Using standard genetic crossing techniques in *C. elegans*, we were able to investigate how these alleles behaved as heterozygotes in the carrier and biallelic state (Fig. 7B). A recessive phenotypic marker (*dpy-5*) was included in the crosses to verify that all progeny analysed were heterozygous. As described above, cilium structure and function assays were performed in an *nphp-4(Δ)* genetic background; controls were also heterozygous for the *dpy-5* allele. The complete genotypes of controls and heterozygotes are shown in Fig. S7.

Using the lipophilic dye-filling assay to assess phasmid cilium integrity, we found that the heterozygous P74S/+ and G155S/+ carrier genotypes showed a modest reduction in dye filling (Fig. 7C,D). By contrast, dye filling was completely abolished in compound heterozygous P74S/G155S worms (Fig. 7C,D). Likewise, assessment of ciliary function via the osmotic avoidance assay revealed grossly normal behaviours for heterozygous P74S/+ and G155S/+ worms, but defective behaviour for compound heterozygous P74S/G155S animals (Fig. 7E). The extent of the osmotic avoidance defect for the compound heterozygous worms was very similar to that of worms homozygous for the P74S allele (Fig. 3G). These data are consistent with the recessive nature of the variants in Joubert syndrome.

## DISCUSSION

### Modelling orthologous JBTS patient missense variants in *C. elegans* identifies differential effects for the P74S and G155S mutations on *mksr-2* (*B9D2*) gene function

In this study, we used CRISPR-Cas9 genome editing in *C. elegans* to model two pathogenic missense variants of *B9D2*/*mksr-2* associated with Joubert syndrome. We found that in the homozygous state, both variants gave rise to cilia and TZ defects that were similar to those from a null allele of *mksr-2*. However, in many assays (dye filling, dendrite length, osmotic avoidance and barrier integrity), the P74S variant phenotype was found to be milder than that of the G155S variant, although both variants showed a less severe overall phenotype compared with the null allele. In line with these findings, super-resolution imaging revealed that the TZ localisation of the G155S variant protein was disorganised, whereas that of the P74S variant was more similar to WT. Together, these findings show that the G155S mutation has a greater effect than the P74S mutation on *mksr-2* gene function.

Our study also shows how *C. elegans* can be used to interpret more complex human genotypes, such as the biallelic P74S/G155S genotype observed in a Joubert syndrome patient (Bachmann-Gagescu et al., 2015). We found that P74S/G155S compound heterozygous worms exhibited more severe phenotypes than heterozygous carriers (P74S/+ and G155S/+), indicating that these alleles are recessive (Fig. 7C-E), which is consistent with the described pedigree for these alleles (Bachmann-Gagescu et al., 2015). Additionally, we found that in heterozygous carriers the WT protein was more efficiently recruited to the TZ than the mutant protein (Fig. 2E), thus providing a mechanism for the recessive nature of these variants. It is unclear why the P74S and G155S mutant proteins are not recruited efficiently to the TZ; one possibility is that the mutations disrupt protein interactions in the B9 complex. Alternatively, the mutations could decrease protein stability. Further experiments are required to assess these hypotheses.

Overall, our work demonstrates the utility of *C. elegans* as a model for interpreting the effects of orthologous ciliopathy patient missense variants on underlying gene function. Also, the modelling of more complex genotypes (e.g. biallelic states) allows for an analysis that accurately reflects more complex patient genotypes.

### Hypomorphic patient alleles provide an opportunity to refine the function of TZ functional modules

In *C. elegans*, most of our knowledge on TZ gene function is derived using null alleles. For example, data from null alleles have established TZ localisation dependencies and assembly hierarchies for *C. elegans* NPHP and MKS module proteins (Bialas et al., 2009; Huang et al., 2011; Jensen et al., 2015; Lambacher et al., 2016; Li et al., 2016; Roberson et al., 2015; Schouteden et al., 2015; Williams et al., 2008, 2011; Winkelbauer et al., 2005; Yee et al., 2015). In the case of the MKS module, a core submodule that includes MKSR-2/B9D2 directs the TZ recruitment of a peripheral submodule, but not vice versa. Therefore, it was surprising to find that in *mksr-2(P74S)* and *mksr-2(G155S)* worms the TZ level and distribution of a core MKS submodule component (MKS-2) were highly disrupted, whereas the TZ localisations of peripheral submodule proteins (JBTS-14/TMEM237 and MKS-6/CC2D2A) were much less affected. The lack of expected correlation for how peripheral and core MKS submodule components localise in *mksr-2(P74S)* and *mksr-2(G155S)* worms indicates that the relationship between these two submodules is more complex than previously thought.

Our data also indicate an especially close functional relationship between MKSR-2 and MKS-2/TMEM216. Strikingly, we have found that MKS-2 redistribution to the proximal end of the TZ in *mksr-2(G155S)* worms is very similar to how the MKSR-2(G155S) protein itself is distributed at the TZ. Thus, TZ redistribution of MKSR-2 causes a comparable redistribution of MKS-2, suggesting a close spatial association of these proteins, perhaps as part of the same protein complex. In support of the latter, the mammalian B9 complex, consisting of MKS1, B9D1 and B9D2, has been shown to be in a larger complex with TMEM216 (Garcia-Gonzalo et al., 2011). However, it has also been found that the B9 complex interacts with CC2D2A (Garcia-Gonzalo et al., 2011; Gupta et al., 2015), which conflicts with our observation of grossly normal MKS-6 localisations in the *mksr-2(G155S)* worms. Further investigation is required to determine exactly how these proteins are organised at the TZ. Nonetheless, our results demonstrate the benefits of using hypomorphic missense alleles to provide new biological insights that cannot be elucidated with null alleles. Therefore, modelling patient missense variants provides an opportunity to gain a better understanding of how the associated proteins contribute to disease mechanisms.

### *C. elegans* as a model to interpret variants of uncertain significance in ciliopathy genes

Increasingly, whole-genome or targeted gene panel sequencing is used to identify candidate pathogenic gene variants in ciliopathies (Bachmann-Gagescu et al., 2015; Wheway et al., 2019). If a candidate pathogenic variant is determined to cause a ciliopathy, this information can be used for genetic counselling, to adjust clinical care based on gene-specific complications and even to qualify patients for gene-specific clinical trials and treatments (Bachmann-Gagescu et al., 2020). Many sequence variants are termed ‘variants of unknown clinical significance’ (VUS) because there are insufficient data for them to be classified as pathogenic or benign (Li et al., 2017). VUS alleles, many of which are missense, are not clinically useful and require further characterisation to be classified as pathogenic or benign. Although *in silico* prediction tools, such as Sorting Intolerant From Tolerant (SIFT) (Sim et al., 2012), MISsense deleTeriousness predICtor (MISTIC) (Chennen et al., 2020), Rare Exome Variant Ensemble Learner (REVEL) (Ioannidis et al., 2016), Combined Annotation-Dependent Depletion (CADD) (Rentzsch et al., 2019) or (Polymorphism Phenotyping (Poly-Phen) (Adzhubei et al., 2010), can predict the pathogenicity of missense variants, these methods are not always reliable and can give contradictory predictions. For example, SIFT, MISTIC and REVEL correctly predict P74S and G155S to be deleterious, whereas CADD predicts that they are likely to be benign. Poly-Phen scores P74S as more damaging than G155S, which is inconsistent with our observations that G155S is the more severe allele.

The data in the present study show that we can successfully model pathogenic B9D2 missense variants in *C. elegans* and determine their precise effects on gene function using quantitative assays of ciliary structure and function. With the exception of ultrastructural analyses, most of these assays are able to distinguish between the severity of the alleles we tested. Therefore, we anticipate that our approach can be applied to the interpretation of missense variation in ciliopathy genes more widely. With regard to the ultrastructural assays, we suspect that our transmission electron microscopical approaches are not sufficiently sensitive to identify subtle alterations in the TZ between strains. Until we can improve the methodology, there are limitations to using transmission electron microscopy for stratifying MKS module gene alleles based on TZ ultrastructure phenotypes.

Interpretation of human missense variations in *C. elegans* is dependent on two key factors: (1) conservation of the mutated residue at the orthologous position in the nematode gene; and (2) shared biology for the human and nematode genes. Ciliopathy genes are very well served by these requirements, with many genes possessing relatively high sequence identity to the worm counterpart and overlapping orthologous functions at the primary cilium (Kim et al., 2018; van Dam et al., 2019). It might also be possible to humanise the nematode by replacing the endogenous gene with its human counterpart (McDiarmid et al., 2018). If the human orthologue retained functionality in the context of the *C. elegans* cilium, this would allow for the testing of a greater number of human variant mutations.

One important caveat when considering *C. elegans* as a tool for interpreting specific human ciliopathy gene variants is cell-, tissue- and organism-specific characteristics in their mechanisms of action. For example, CEP290 regulates the TZ recruitment of NPHP1/4 in mammalian cells but not in *C. elegans*; furthermore, although individual MKS and NPHP module genes are mostly dispensable for cilium formation in nematodes, several of these genes are partially required for ciliogenesis in mammals, at least in some tissue and cell types (Lewis et al., 2019; Wiegering et al., 2018). Also, vertebrates possess two RPGRIP1-like genes (RPGRIP1 and RPGRIP1L), which interact functionally in a tissue-specific manner to regulate TZ assembly differentially (Wiegering et al., 2018). Thus, TZ findings from sensory neuronal cilia in worms might not always be applicable directly to all types of cilia and associated molecular pathways in vertebrates. Nevertheless, the highly conserved nature of many aspects of the TZ molecular pathways, including genetic interaction between MKS and NPHP modules (Yee et al., 2015), provides confidence that gene variant interpretation in *C. elegans* can be extrapolated to the mammalian orthologues in many cases. As further proof of principle towards this goal, it will be instructive to assess whether human TZ genes can retain functionality in ‘humanized’ worms.

### Conclusions

This study acts as a proof of concept that known pathogenic missense variants in a TZ ciliopathy gene can be recapitulated and interpreted in worms. Modelling ciliopathy patient variants in *C. elegans* has the potential to be a powerful diagnostic tool as there is a move towards personalised medicine for patients with rare genetic disorders. As shown here, patient variants can be generated efficiently in *C. elegans* using CRISPR-Cas9 technology and identified using the simplified PCR-based allele detection strategy that we developed (Fig. S1). Indeed, with streamlined methodologies, it should be possible to generate many alleles in a relatively short time frame at relatively low cost and manpower compared with other multicellular systems. The workflow to generate and characterise ciliopathy-associated variants described here can also be extended to other conserved cilia genes and ciliopathies. In conclusion, the efficiency of genetic engineering and relatively simple quantitative assays to characterise ciliary structure and function in *C. elegans* make it an excellent system for interpreting hypomorphic and VUS alleles associated with ciliopathies.

## MATERIALS AND METHODS

### *C. elegans* strains and maintenance

All *C. elegans* strains were maintained at 15°C or 20°C on nematode growth medium (NGM) agar plates seeded with OP50 *Escherichia* *coli* using standard worm culturing techniques (Brenner, 1974). PCR was used to confirm the genotypes of all strains. Young adult hermaphrodites were synchronised by selecting L4 larvae and incubating at 20°C for 16-20 h before imaging or phenotypic characterisation. A list of worm strains used in this study can be found in Table S2.

### Knock-in of mNG using CRISPR-Cas9

mNG is licensed by Allele Biotechnology and Pharmaceuticals (Shaner et al., 2013). A plasmid (dg353) containing *C. elegans*-optimised mNG (Hostettler et al., 2017) was obtained from Dominique Glauser (University of Fribourg, Switzerland). High-fidelity PCR was used to add a 12aa flexible linker (GTGGGGSGGGGS) to the N- and C-termini of mNG. PCR products were then cloned into pJET1.2 using the blunt end CloneJET PCR Cloning Kit (Thermo Scientific). Two rounds of high-fidelity PCR were used to generate linear mNG repair templates with 35 bp homology arms as previously described (Paix et al., 2015). All CRISPR protocols used Alt-R Cas9 nuclease 3NLS (IDT, #1074181), Alt-R tracrRNA (IDT, #1072533) and custom-generated gene-specific Alt-R crRNA from IDT. Gene-specific crRNA sequences were chosen based on proximity to the desired edit and the Azimuth 2.0 score (Doench et al., 2016). All RNA was reconstituted with 5 mM Tris-HCl pH 7.5 and stored at −80°C. Single-stranded donor oligonucleotide (ssODN) repair templates were ordered as standard desalt DNA oligonucleotides from Sigma-Aldrich, reconstituted with 1 M Tris pH 7.4, and stored at −20°C. A list of guide sequences and repair template sequences can be found in Table S3. All CRISPR injection mixes were prepared on ice, mixed gently, centrifuged at ∼15,000 ***g*** for 2 min, and incubated at 37°C for 15 min before injection. N-terminal or C-terminal knock-ins of mNG were performed using a *dpy-10* co-CRISPR strategy (Paix et al., 2015). mNG knock-in injection mixes were as follows: 1 μl gene-specific crRNA (0.3 nmol/μl), 1 μl tracrRNA (0.425 nmol/μl), 0.2 μl *dpy-10* crRNA (0.6 nmol/μl), 0.23 μl *dpy-10* ssODN (500 ng/μl), 5-6 μl mNG repair template (up to a maximum final concentration of 500 ng/μl), 1-2 μl 1 M KCl, 0.38 μl HEPES (200 mM, pH 7.4), 0.2 μl Cas9 (61 μM), for a final volume of 10 μl. All Dpy and Rol F1 were screened in pools of eight hermaphrodites. The knock-in efficiency of mNG varied from 0.1% to 2%, with an average of 800 F1 screened per gene (range 376-2408). Sanger sequencing confirmed the accuracy of all knock-ins. Using this approach, we generated *mks-2(oq101[MKS-2::mNG])*, *cep-290(oq103[CEP-290::mNG])*, *mksr-2(oq108[mNG::MKSR-2])*, *nphp-4(oq109[mNG::NPHP-4])*, *mks-5(oq112[mNG::MKS-5])*, *jbst-14(oq127[mNG::JBTS-14])* and *mks-6(oq128[MKS-6::mNG])*. A dye-filling assay was used to confirm that the mNG tag did not appreciably disrupt the function of the associated genes, with the exception of a small effect on *mks-2* (Fig. S6A).

### Engineering missense *mksr-2* variants with CRISPR-Cas9

The patient variants, *mksr-2(oq108,oq125[mNG::MKSR-2(P74S)])* and *mksr-2(oq108,oq126[mNG::MKSR-2(G155S)])*, were generated using ssODN repair templates, *unc-58* co-CRISPR (Arribere et al., 2014) and RFLP analysis to detect the variant (Paix et al., 2017). We modified the *unc-58* ssODN to include two additional silent mutations in the guide sequence (Table S3). *mksr-2(oq108[mNG::MKSR-2])* worms were used for the injections. crRNA and ssODN were reconstituted as described in the previous subsection. Injection mixes were prepared on ice as follows: 1 μl P74S crRNA (0.3 nmol/μl), 1 μl G155S crRNA (0.3 nmol/μl), 1 μl tracrRNA (0.425 nmol/μl), 0.25 μl *unc-58* crRNA (1 nmol/μl), 0.25 μl *unc-58* ssODN (500 ng/μl), 0.5 μl P74S ssODN (1 μg/μl), 0.5 μl G155S ssODN (1 μg/μl), 2 μl 1 M KCl, 0.38 μl HEPES (200 mM, pH 7.4), 0.2 μl Cas9 (61 μM), and RNAse-free water up to 10 μl. The injection mix was incubated at 37°C for 15 min before injection. All Unc F1 were screened in pools of between one and four hermaphrodites. The P74S and G155S variants introduced a restriction enzyme cut site in the *mksr-2* gene (BstUI and NheI, respectively). Using this restriction enzyme screening technique, the P74S and G155S variants were generated at an efficiency of 3.1% and 4.9%, respectively. The *mksr-2(oq137[P74S])* and *mksr-2(oq138[G155S])* strains were generated with the same approach and repair templates, except that WT N2 worms were used for the injections. These variants were detected in the F1 with a simplified PCR-based approach using a primer that binds specifically to the engineered silent and missense mutations (Fig. S1). Both variants were generated with an efficiency of 12.5%. Sanger sequencing confirmed the accuracy of all CRISPR-generated alleles, and each strain was outcrossed twice before analysis.

### Wide-field epifluorescence live imaging and quantification

Worms were immobilised in 40 mM tetramisole (Sigma-Aldrich) on 4% or 10% agarose pads. All images were acquired on an upright Leica DM5000B epifluorescence microscope. Images were collected with ×100 (1.40 NA) or ×63 (1.3 NA) oil-immersion objectives and captured with an Andor iXon+ camera run by Andor software. All image analysis and preparation was performed with FIJI/ImageJ (National Institutes of Health). The TZ length of the mNG::MKSR-2 or mNG::MKS-5 signal length in phasmid neurons was calculated using a ‘full width at half max’ approach from line scans at the centre of the TZ from single focal planes. The fluorescence levels of mNG-taggged proteins at the TZ were quantified from a single focal plane where both TZs were in focus. The integrated signal intensity in a 40×40 pixel box around the TZ pair was measured and then increased by one pixel in each direction. The integrated signal intensity of this 42×42 pixel box was used to calculate and subtract background fluorescence. Lengths of phasmid cilia and dendrites were measured from maximum projections of XBX-1::tdTomato using the segmented line feature of ImageJ. The span of the amphid BBs were measured from maximum projections with a box that was drawn around the BBs. To measure RPI-2::GFP levels in the cilium, a segmented line was drawn along the cilium in the XBX-1::tdTomato channel from the base to beyond the tip of the cilium. RPI-2::GFP levels 4 µm from the base were used in calculations. The background was calculated by averaging the fluorescence intensity values at the end of the line beyond the tip of the cilium. TRAM-1::tdTomato was quantified in a similar manner, except TRAM-1 levels were measured 2-4 µm from the base of the cilium.

### Confocal super-resolution microscopy

Confocal-based super-resolution microscopy was achieved with a reduced pinhole size and deconvolution (Lam et al., 2017). Super-resolution imaging was performed on an Olympus FV3000 confocal laser scanning microscope with a ×60 (1.40 NA) oil-immersion objective and high-sensitivity spectral detector using FluoView Olympus software. The Airy disk pinhole size was set to 0.4 (81 µm), and ×15 optical zoom was used. The pixel size was 26.7 nm, with an *x-y* optical resolution of 148.6 nm. *Z*-stacks were acquired with 0.2 µm steps. All images were deconvolved with Olympus cellSens software using default values. Imaris v.7.6.5 was used to generate 3D models. To account for the lower *z*-plane resolution, a step size of 0.1 µm was used to create the 3D models.

### Assays to characterise ciliary defects: dye filling, roaming and osmotic avoidance

All phenotypic characterisation of ciliary defects was performed as previously described (Sanders et al., 2015), with young adult hermaphrodites. Dye-filling assays were performed with worms that were incubated in a 1:200 dilution of DiI or DiO (Invitrogen) in M9 for 60 min then allowed to recover for 30 min on seeded NGM plates. Worms were then mounted on 4% agarose pads with 40 mM tetramisole (Sigma-Aldrich) to assess dye uptake with a wide-field epifluorescence microscope. DiI (red) was used for image acquisition and DiO (green) was used for quantification. The assay was repeated at least three times, with 30-50 worms per trial. Roaming assays were performed with a single worm that was placed on a fully seeded NGM plate for 20 h at 20°C. The worm was removed, the plate was placed on a 5×5 mm grid, and the number of squares that the worm entered was counted. For each strain tested, the assay was repeated at least three times, with 15-20 worms per trial. Dye-filling and roaming assays were performed blindly by randomizing the strains before the start of the experiment. For the osmotic avoidance assay, worms were placed on an unseeded NGM plate for 2 min before the assay. Six worms were placed in a high-osmolarity ring (8 M glycerol with Bromophenol Blue) and observed for 10 min. The time when any worm escaped the barrier was noted, and the worm was removed from the assay. Three independent trials were performed for each osmotic avoidance assay.

### Transmission electron microscopy

Young adult hermaphrodites were fixed, embedded, sectioned and imaged as previously described (Sanders et al., 2015). Briefly, worms were fixed in 2.5% glutaraldehyde in Sørensen's phosphate buffer (0.1 M, pH 7.4) for 48 h at 4°C. Samples were post-fixed in 1% osmium tetroxide for 1 h and dehydrated through an increasing ethanol gradient. Ethanol was substituted for propylene oxide before EPON resin embedding overnight. Serial, ultra-thin (90 nm) sections were cut using a Leica EM UC6 Ultramicrotome, collected on copper grids and subjected to double contrasting using 2% uranyl acetate for 20 min followed by 3% lead citrate for 5 min. Imaging was performed on a Tecnai 12 (FEI software), with an acceleration voltage of 120 kV.

### Statistical analysis

All graphs and statistical analyses were performed in Microsoft Excel with the Real Statistics Resource Pack v.7.2 (www.real-statistics.com). The Shapiro–Wilk test was used to determine whether the data were normally distributed. One-way ANOVA followed by Tukey's post hoc test or the Kruskal-Wallis test followed by Dunn's post hoc test were used to calculate *P*-values for multiple comparisons. Differences were considered significant when *P*<0.05.

## Supplementary Material

Supplementary information
